# Quantitative Assessment of PM_2.5_ Sources and Their Seasonal Variation in Krakow

**DOI:** 10.1007/s11270-017-3483-5

**Published:** 2017-07-21

**Authors:** Lucyna Samek, Z. Stegowski, L. Furman, K. Styszko, K. Szramowiat, J. Fiedor

**Affiliations:** 1 Faculty of Physics and Applied Computer Science, AGH University of Science and Technology, Al. Mickiewicza 30, 30-059 Krakow, Poland; 2 Faculty of Energy and Fuels, Department of Coal Chemistry and Environmental Sciences, AGH University of Science and Technology, Al. Mickiewicza 30, 30-059 Krakow, Poland

**Keywords:** Particulate matter, Energy dispersive X-ray analysis, Ion chromatography, Positive matrix factorization

## Abstract

In industry areas of Poland such as Silesia or urban sites like Krakow and some other cities, the levels of pollutants frequently breach air quality standards. Particulate matter (PM) is the most important constituent of atmospheric pollution. Beginning on 1st February 2014 until 31st January 2015, the samples of fine particulate matter PM_2.5_ (aerodynamic diameter of particles less than or equal to 2.5 μm) were collected at a site in the south-eastern Krakow urban background area. During this period, 194 samples were taken. The samples showed daily variation of PM_2.5_ concentration. From these data, monthly variations were estimated and presented in this paper. Monthly integrated data are more representative for the Krakow urban background and show seasonal variation of PM_2.5_ pollution. The lowest monthly concentration value was found for August 2014—about 10 μg m^−3^, the highest for February 2014–70 μg m^−3^, whereas the average annual value was about 31 μg/m^3^. Utilizing X-ray fluorescence method, concentrations of 15 elements for each sample were determined and 8 inorganic ions were analyzed by ion chromatography. Additionally, the samples were analyzed for black carbon (BC). Receptor model PMF (positive matrix factorization) was used for source identification and apportionment. The modeling identified six sources and their quantitative contributions to PM_2.5_ total mass. The following sources were identified: combustion, secondary nitrate and sulfate, biomass burning, industry or/and soil and traffic. Finally, monthly variations of each source are presented.

## Introduction

Outdoor air pollution and especially the fine fraction of particulate matter (PM) have been associated with a variety of adverse health effects. High levels of PM with aerodynamic diameter less than 2.5 μm (PM_2.5_) cause a serious risk for public well-being: respiratory diseases such as asthma attacks and acute bronchitis or cardiovascular complications (Silva et al. 2013; Puett et al. 2014). In many European cities, PM_2.5_ annual average concentration exceeds limit value of 25 μg m^−3^ given by the European Directive 2008/50/EC. Knowledge of the major contributing emission sources to PM_2.5_ is needed in order to evaluate the potential risks of specific sources for human health and to support local authorities in limiting the impact of pollutants. Apart from average, the seasonal variations of PM, elemental concentrations variability and chemical content which determines harmfulness of particulates, are exceptionally important in air quality assessment.

In the previous study, Samek (Samek 2016) applied Air Quality (AirQ) software model for toxicity of particulate matter determination. Total mortality due to the exposure to PM_10_ was 41 deaths per 100,000 inhabitants in 2005 and dropped to 30 in 2013, while keeping cardiovascular mortality two times lower then total mortality. Hospital admissions due to respiratory diseases were more than an order of magnitude higher than the number of respiratory mortalities. Krakow is the second largest city in Poland with 760,000 inhabitants, situated in a valley—a semi-closed basin. Inconvenient geographical location, large number of domestic coal and biomass stoves and other local pollution sources, including heavy traffic give rise to increased levels of particulate matter. Samek (2012), Samek et al. (2013) have recently reported PM_10_ concentrations of 15–45 μg m^−3^ during summer and 50–350 μg m^−3^ during winter time when domestic heating becomes an important pollution source. This study reports the annual average concentration of PM_2.5_ in Krakow about 30 μg m^−3^ and more than 100 μg m^−3^ during over a dozen days in the year. It is natural to ask what the major pollutants are: traffic, industry, biomass burning/residential activities, soil dust, sea salt, or other.

This article describes the source apportionment results for PM_2.5_ together with monthly/seasonal differences in the source contributions. A positive matrix factorization (PMF) receptor model, based on the elemental, inorganic ion and Black carbon data was used to identify and apportion potential emission sources of the study area. Paatero and colleagues developed the PMF model for aerosol applications (Paatero and Tapper 1993, 1994; Paatero 1997). Based on chemical profiles at the receptor site, the method provides the relative contributions of different pollution sources. Recently, a large number of papers considering source identification and apportionment has appeared (Viana et al. 2008; Karagulian et al. 2015; Amato et al. 2016; Manousakas et al. 2015, 2017). The previous paper (Samek et al. 2017) contains application of PMF modeling for source identification and apportionment. The input for modeling were elemental and black carbon analyses.

For the purpose of this study, a year-long PM_2.5_ sampling campaign, from February 2014 to January 2015 was carried out at Krakow urban background site. Samples were collected on every second day (in February on a daily basis) giving finally 194 samples for analysis. Elemental content was determined using ED-XRF (Energy–Dispersive X–ray Fluorescence) spectrometry, inorganic ions were identified via ion chromatography and additionally, black carbon concentration was determined. In order to observe seasonal variability of the PM_2.5_ pollution and ensure that the results could be more representative for the entire Krakow urban background area, monthly variations were estimated and presented in this paper. Based on the sample species (elements, ions and BC) the PMF modeling was applied for source identification and apportionment. Six sources have been identified and their seasonal contributions to PM_2.5_ total mass, reconstructed by the PMF, are presented.

## Experimental

### Study Area and Sampling

**Fig. 1:**
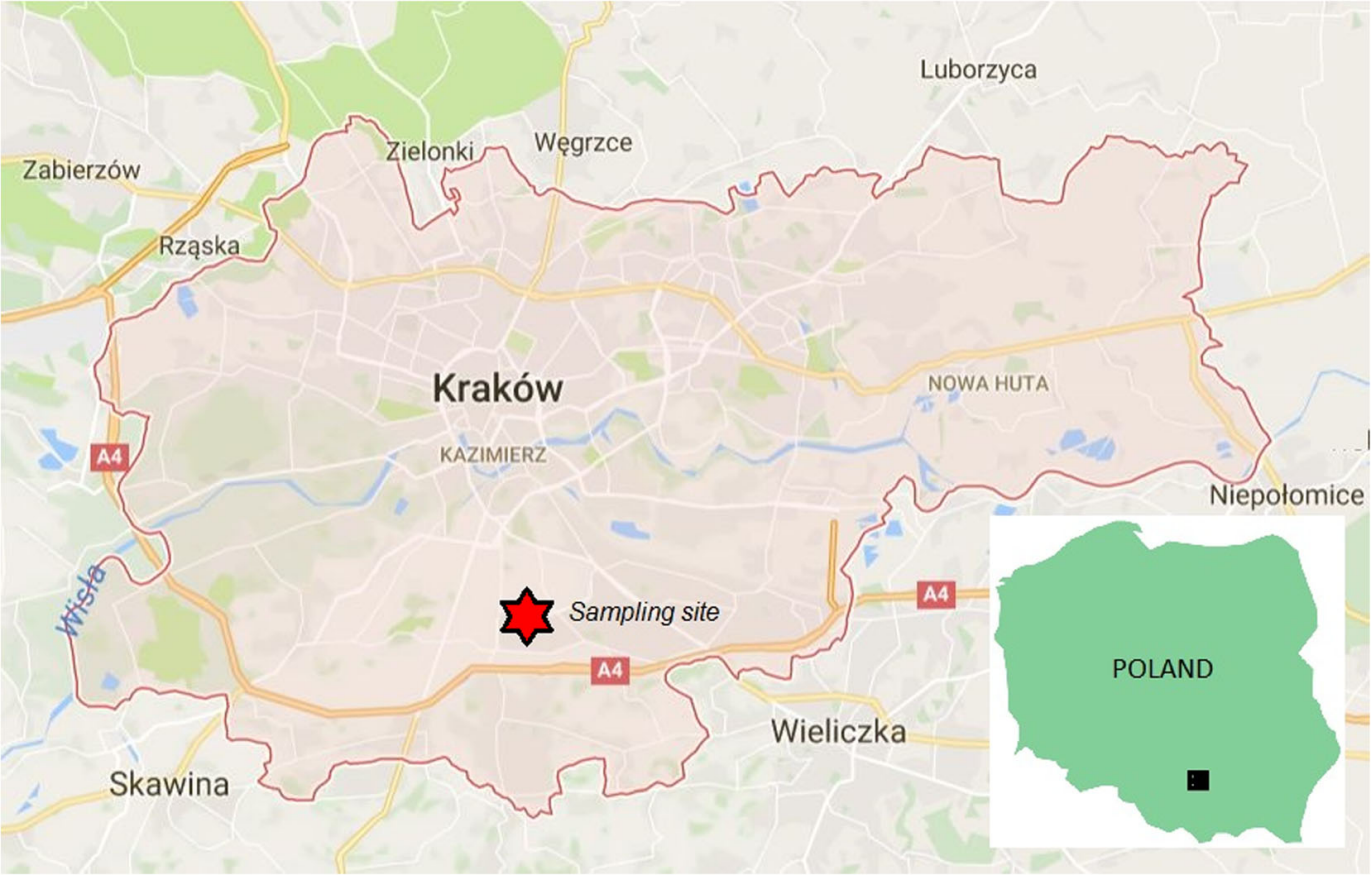
Location of sampling site

Measurements were performed by the Voivodship Inspectorate of Environmental Protection in Krakow at an urban background site, settled in a high-density residential zone, in the south-eastern part of Krakow (see map in Fig. 1). Twenty-four-hour PM_2.5_ samples were collected on quartz filters QMA Whatman (47 mm), using a low volume sampler (flow rate 2.3 m^3^/h). Height of the sampler was 2.5 m. Filters were stored at temperature 20±1C and RH 50±% for 48 h before weighing.

The major local sources of pollution are combustion, biomass burning and traffic. There are the following emitters of pollutants in Krakow: in the northern part of the city the Huta Alcellor Mittal steel works, the Cementownia cement factory, the EC Krakow coal-fired power plant, numerous small coal-fired low efficiency boilers distributed over the city and traffic through the city (Junninen et al. 2009).

### Chemical Analysis

#### Elemental Concentrations

Concentrations of the following elements were quantified: Cl, K, Ca, Ti, V, Cr, Mn, Fe, Ni, Cu, Zn, Br, Sr, Rb, and Pb. Samples of PM_2.5_ were analyzed as thin samples, using a multifunctional energy dispersive X-ray fluorescence spectrometer. The instrument is equipped with a capillary X-ray micro-beam optics, a broad X-ray beam from a molybdenum secondary target for XRF analysis of bulk samples and a beam for total reflection X-ray technique. Two kilowatts of molybdenum tube is the source of X-rays. The excited X-rays were detected by a Si(Li) detector with resolution of 170 eV at energy of 5.9 keV. The detector is cooled by liquid nitrogen; active area 30 mm^2^, thickness 3 mm (be window thickness is equal to 0.025 mm).

Data collection was accomplished using the Canberra system (http://www.canberra.com). The measurements were carried out under the following conditions: voltage of 55 kV, current of 30 mA, measuring time of 10,000 s, and under atmospheric air. In order to calculate concentrations of different elements in the filters, the spectrometer was calibrated using thin-film standards (Micromatter, USA). The calibration was verified by the analysis of NIST Standard Reference Material 2783 (air particulate matter on filter media). The XRF spectra were quantitatively analyzed utilizing the QXAS package (Vekemans et al. 1994). Detection limits are shown in Table 1.

**Table 1:** Detection limits of chemical species

Species	Detection limits [ng m^−3^]
Cl	152
K	23
Ca	7.5
Ti	5.7
Cr	2.2
Mn	5.2
Fe	3.1
Ni	1.1
Cu	2.7
Zn	2.1
Br	1.1
Pb	2.3
Cl^−^	90
Na^+^	90
K^+^	40
Ca^2+^	310
NH_4_^+^	70
NO_3_^−^	40
SO_4_^2−^	20
PO_4_^3−^	80

#### Inorganic Ions Concentrations

Concentrations of the following inorganic anions and cations were analyzed with isocratic ion chromatography: Na^+^, K^+^, Mg^2+^, Ca^2+^, NH_4_^+^, NO_3_^−^, Cl^−^, and SO_4_^2−^. Two circular filter aliquots (ø 8 mm) per sample were extracted under ultrasonic agitation for 20 min, in either 1.5 mL of extra pure water or in 1.5 mL of the 12 mM methanesulfonic acid (MSA) in order to determine concentrations of anions and cations, respectively.

Ion chromatography (IC) analysis was performed on an ICS-1100 instrument (Thermo Scientific) equipped with an autosampler AS-DV and ion-exchange columns:

- Ion Pac AS22 (4 × 250 mm) for anions, mobile phase: 4.5 mM Na_2_CO_3_ + 1.4 mM NaHCO_3_,
- CS16 (5 × 250 mm) for cations, mobile phase: 12 mM MSA.

After electrochemical suppression (AERS 500 (4 mm) and CERS 500 (4 mm) suppressors), quantification was performed with a conductivity detector. The injection volume was 25 μL and the flow rate 1.2 mL min^−1^.

Calibration was performed against external standards diluted from stock solutions supplied by Thermo Scientific. The limit of detection (defined as ×3 standard deviation for the field blank samples) of the method for atmospheric samples is presented in Table 1.

#### Concentrations of Black Carbon

Black carbon was determined by UV-Vis spectroscopy. Spectroscopic measurements were performed using Varian Cary 50-Bio UV-Vis spectrophotometer (Agilent). Transmittance was recorded at the 880 nm wavelength, in at least triplicate for each sample. Additionally, transmission spectra were collected in the range of 200–1000 nm. All measurements were carried out in reference to air. Concentrations of BC were calculated according to formulas listed by Quincey (Quincey 2007). Transmittance at 880 nm was measured for clean filter (*I*_0_) and the sample (*I*), then absorption coefficient was calculated from the formula *α* = A/V lnI_0_/I [1/m]; where:

$$ \begin{array}{l}\mathrm{A}-\mathrm{sample}\kern0.5em \mathrm{area}\left[{\mathrm{m}}^2\right],\\ {}\mathrm{V}-\mathrm{volume}\left[{\mathrm{m}}^3\right].\end{array} $$

Finally *C*_BC_ = 6.02 × 10,000 × *α* [μg/m^3^], where *C*_BC_ concentration of black carbon is in μg/m^3^.

### Positive Matrix Factorization

Apart from the bulk particulate matter (PM) and the component (elements and ions) analysis, identification of the sources is the main goal of the current investigation. Positive matrix factorization (PMF) receptor model, widely used at present in many countries (Karagulian and Belis 2012), has been utilized.

PMF receptor model solves the set of equations:

1 $$ {x}_{ij}=\sum_{k=1}^p{g}_{ik}{f}_{kj}+{e}_{ij} $$

where *x*_*ij*_ is an element of concentration matrix *X* (*i*—sample index and *j*—species index), *g*_*kj*_ is an element of source contribution matrix *G* with *p* sources (*k* is sources index), *f*_*kj*_ is an element of *F* source profile matrix and finally *e*_*ij*_ is an element of residual matrix *E* (Manousakas et al. 2015). PMF multivariate statistical method decomposes the concentration matrix (*X*) to source contribution (*G*) and source profile (*F*) matrixes in such a way that *G* and *F* obtain non-negative values only, ensuring physical meaning of the model.

For PMF modeling the EPA PMF 5.0 software, developed by the United States Environmental Protection Agency (US EPA), has been used. As an input, not only concentration matrix (*X*) but also appropriate uncertainty matrix (*U*) is required. For a given number of factors (*p*) the matrixes *G* and *F* are adjusted by minimizing the objective function *Q* which is defined as:

2 $$ Q=\sum_{j=1}^m\sum_{i=1}^n\frac{e_{ij}^2}{u_{ij}^2} $$

where *u*_*ij*_ is an element of uncertainty matrix (*U*), *m* is the number of species and *n* is the number of samples. A general optimization method where measured value is “weighted” by its uncertainty requires the accurate uncertainty estimation. In the present work, if the concentration is less than or equal to the detection limit (DL) for a given element, the uncertainty is set at 5/6 DL and the concentration replaced by ½ DL (Pollisar A.V., 1998). Missing data was substituted by median values and the corresponding uncertainties were set at four times the median value. In addition, PMF 5.0 software calculates signal to noise ratios (S/N) for each chemical component (PMF 5.0 Help) in order to classify species as “strong,” “weak,” or “bad.” The species characterized as “bad” are removed from factorization. As mentioned before, the S/N ratio is a basic criterion of classification; however, final decision depends on factorization results and ability to tell sources apart. After factorization run, PMF software gives possibility to analyze the factorization stability by the “Fpeak Bootstrap Method” (PMF 5.0 Help; Manousakas et al. 2017).

Number of factors assumed (*p*) depends on what form the data sets appear (number and type of chemical components), analysis of the physical sources of particulate matter in the investigated area and the ability of their identification by factorization.

In the presented study, many PMF modeling runs were performed with diverse combination of data sets and number of factors. For each model a statistical analysis has been done and an ability of sources identification was studied. Finally, six factors emerged and the following data were classified as “strong”: NO_3_^−^, NH_4_^+^, Cl^−^, SO_4_^2−^, BC, PO_4_^3−^, Na+, K+, Fe, Zn, Pb, Br, Rb, “weak”: Ca^2^+, Ti, Cr, Mn, Cu, Sr, and “bad”: Ni, V, Cl, K, Ca. PM2.5 bulk concentration was set as a “total variable.”

## Results and Discussion

### Elemental and Ions Analyses

**Fig. 2:**
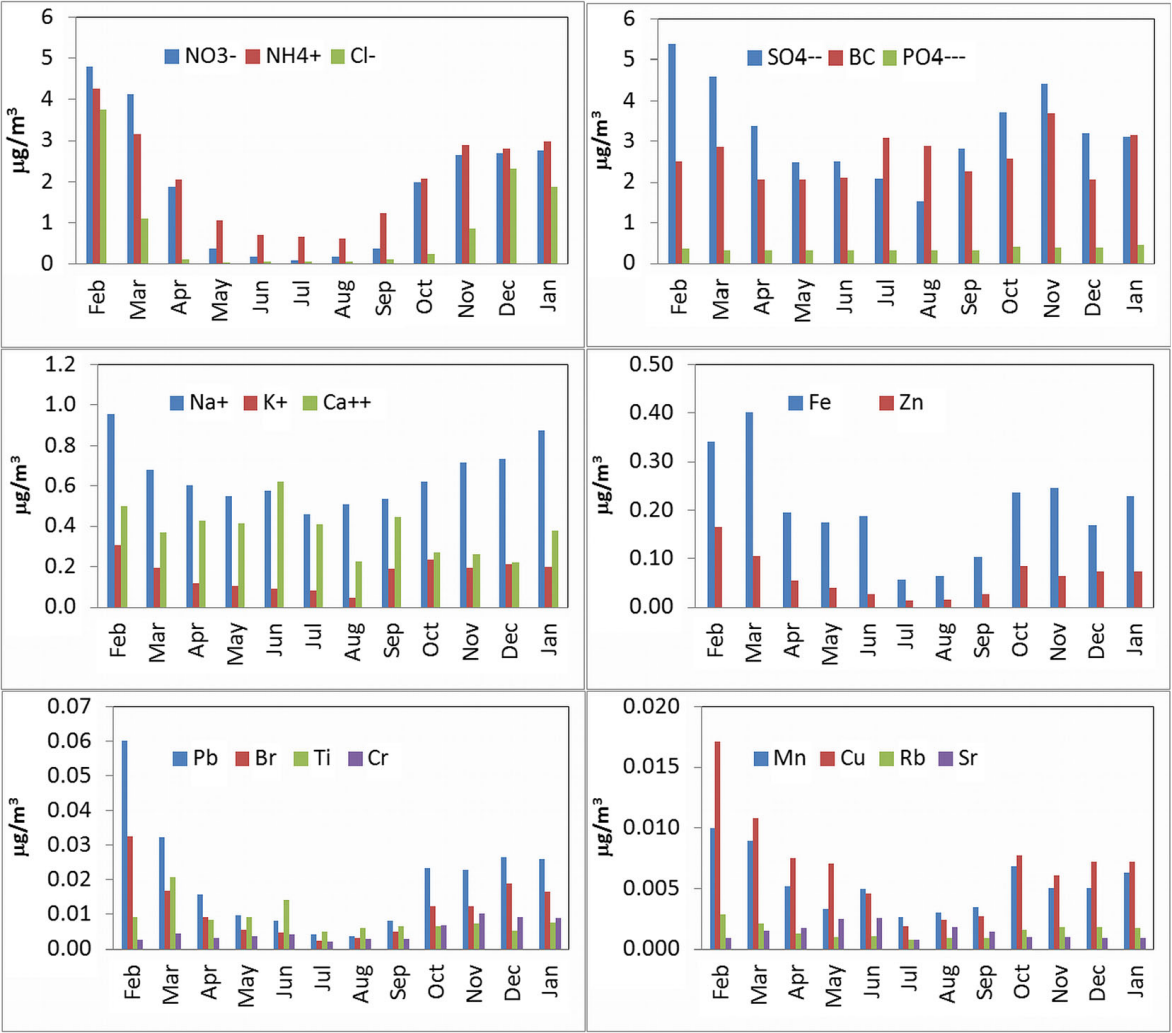
Monthly elemental and ion concentrations

Monthly variation of major chemical species of PM_2.5_ (NO_3_^−^, NH_4_^+^, Cl^−^, SO_4_^2−^, PO_4_^3−^, Na^+^, K^+^, Ca^2+^, Fe, Zn, Mn, Cu, Rb, Sr, Pb, Br, Ti, Cr, and BC) is shown in Fig. 2. Concentrations of NO_3_^−^, NH_4_^+^, Cl^−^, SO_4_^2−^, Na^+^, K^+^, Fe, Zn, Mn, Cu, Pb, and Br show seasonal differences with higher values observed in the winter season. Analytical results illustrated that in February 2014 concentrations of Cl^−^ and K^+^ reached the values as high as 3.8 and 0.30 μg/m^3^, respectively. Moreover, the concentrations of nitrates (4.8 μg m^−3^) and sulfates (5.4 μg m^−3^) were also very high, however they have fallen to 0.07 and 1.5 μg m^−3^, respectively, in August 2014. Interestingly, the highest concentrations of Ca^2+^, Ti, and Sr were observed in March (370, 21, and 2 ng m^−3^) and June 2014 (680, 14, and 3 ng m^−3^, respectively). On the other hand, the lowest concentrations of Fe, Zn, and Cu were detected in July, August, and September 2014. High concentrations of Cr observed between October 2014 and January 2015 have reached 18 ng m^−3^ on certain days. Unlike previously commented chemical species the time series of BC concentrations was flat, in the range of 2.1–3.7 μg m^−3^.

Average annual concentrations of PM_2.5_ chemical species in Krakow and six other cities are presented for comparison in Table 2. The mean PM_2.5_ concentration in Krakow (31 μg m^−3^) was twice as large as that in Genoa, Barcelona, Florence, and similar to Milan. The highest PM_2.5_ mass concentration of 104 μg m^−3^, on average, was observed in Shanghai. The concentration of Cl in Krakow was much higher than that found in Genoa, Barcelona, Florence, and Milan but it was half of that in Shanghai. The concentrations of all analyzed species in Shanghai and Beijing were much higher than the concentrations in the remaining cities. K, Ca, and Fe concentrations were two times higher in Krakow than in Genoa. As shown in Table 2, the BC concentration was higher in Krakow than in Barcelona and Florence by a factor of 3 whereas comparable to Milan. Also, the Zn concentration reached much higher values in Krakow than in Genoa, Florence, and comparable to that in Milan. Astonishingly, the lowest values of Ni and V concentrations were detected in Krakow, Barcelona, and Florence. Concentration of sulfates was two times higher in Krakow than in Barcelona, Florence, and Milan. Concentration of ammonium in Krakow was similar to that in Milan and three times higher than in Barcelona and Florence. Nitrates had higher concentration in Milan than in Krakow whereas the lowest value of nitrates was observed in Florence. It is worth noting that we compared extreme values only, being aware that we put side-by-side different sampling years with possible distinct weather conditions and diverse periodic trends at the cities.

**Table 2:** Yearly mean concentrations and standard deviations of species in Krakow and other cities (elemental and ion concentration [ng m^−3^], PM_2.5_ [μg m^−3^])

Species	Krakow 2014	Barcelona 2013^a^	Florence 2013^a^	Genoa 2009–2010^b^	Milan 2013^a^	Shanghai 2009–2010^c^	Beijing 2010 ^d^
PM2.5	31 ± 30	15	13.2	15 ± 7	30	104	
Cl	1349 ± 2600	178	22	57 ± 74	272		2204
K	226 ± 410	87	172	120 ± 70	303	2253	2222
Ca	134 ± 250	143	104	68 ± 45	237	3407	1048
Ti	8.9 ± 8.7	4.9	3.5	6 ± 4	14.3		36.2
V	1.7 ± 1.3	3.7	1.7	12 ± 14	2.6		15.3
Cr	4.9 ± 4.0	1.3	0.8	3 ± 2	2.1	31	22.4
Mn	5.7 ± 6.0	4.6	2.1	4 ± 3	8.5	132	62
Fe	210 ± 190	146	88	135 ± 65	317	2381	1080
Ni	1.2 ± 0.9	1.3	1	7 ± 7	4.6	27	28.1
Cu	7.6 ± 8.5	6.9	88	5 ± 2	32.6	29	36.8
Zn	69 ± 71	45	10.3	17 ± 10	56.2	465	313
Br	13 ± 14		3.6	4 ± 2	10.3		60.2
Rb	1.6 ± 1.1	0.2	0.7	4 ± 2	0.9		
Sr	1.7 ± 5.6	0.6	0.8		13.5	212	
Pb	23 ± 25	5.4	3.7	8 ± 11	15.2	133	117
BC	2600 ± 1200	1000	930		1780		
Cl^−^	1007 ± 1300						
Na^+^	820 ± 390						
K^+^	170 ± 90						
Ca^2+^	390 ± 250						
NH_4_^+^	2180 ± 1500	709	548		2201		
NO_3_^−^	2040 ± 1700	922	639		5675		
SO_4_^2−^	3400 ± 1500	1685	1542		1934		
PO_4_^3−^	350 ± 50						

^a^Amato et al. (2016)

^b^Cuccia et al. (2013)

^c^Wang et al. (2013)

^d^Yu L et al. (2013)

### Source Apportionment Results

**Fig. 3:**
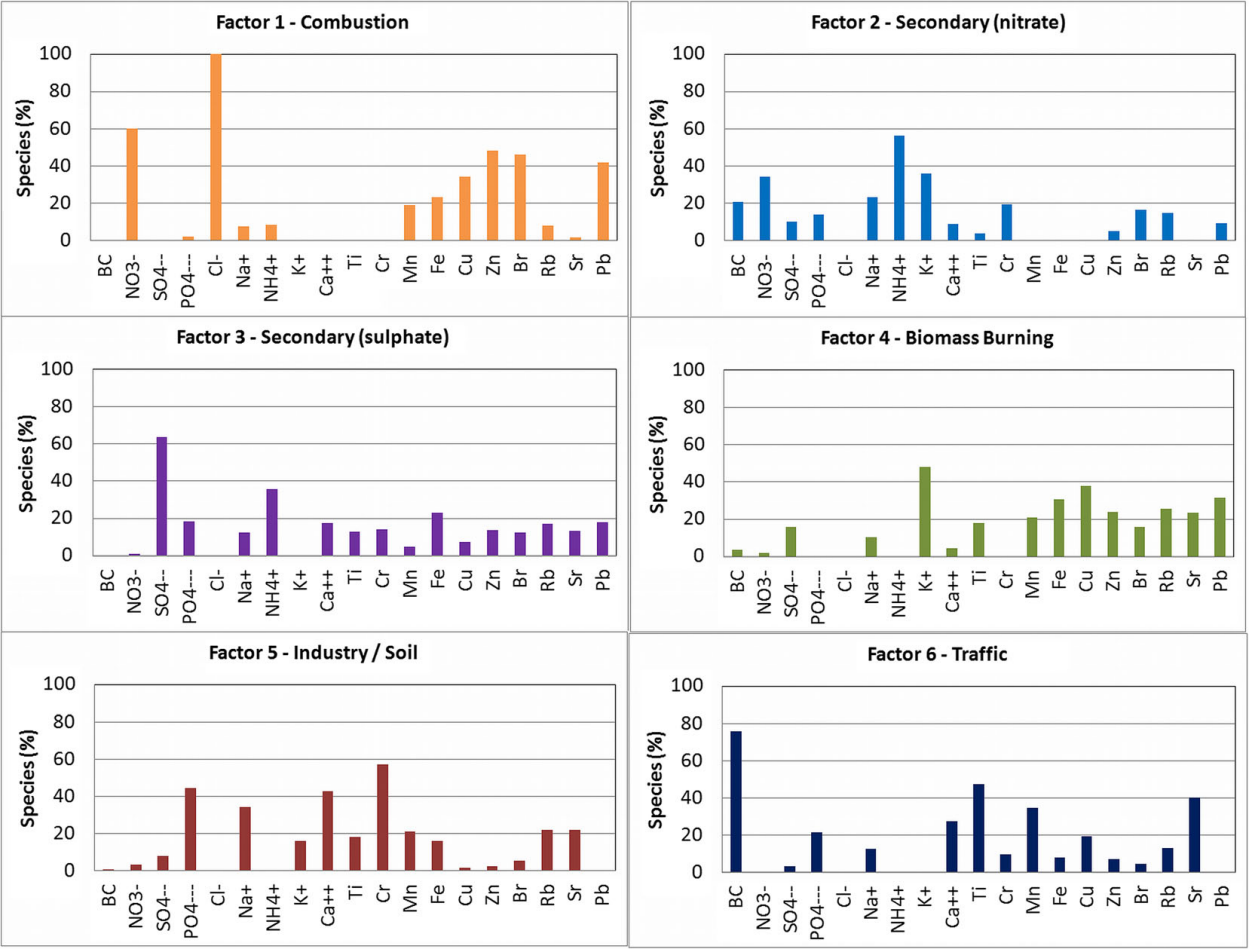
Factor profiles from PMF modeling

PMF modeling for six factors gave the profiles which are presented in Fig. 3.

Since for the first factor the main tracers are Cl^−^, NO_3_^−^, Zn, Br, Pb, and Cu, it was identified as combustion source. It can be associated to combustion of coal (Cl^−^) (Kabata-Pendias and Pendias 1999; Almeida et al. 2005), waste (Cu, Mn, Pb, Zn, Cl, Br) (Chueinta et al. 2000; Vallius et al. 2005) as well as emission from motor vehicles (NO_3_^−^,Cu, Zn, Pb) (Rogula-Kozlowska et al. 2011).

The second factor was identified as secondary nitrate source where the main tracers are NH_4_^+^, K^+^, Na^+^, and NO_3_^−^. Jeong presented main indicators of secondary nitrate as NH_4_^+^, NO_3_^−^, and Cl^−^ (Jeong et al. 2011). Ammonium nitrate is formed from NOx precursors emitted from traffic, biomass burning and industries (Amato et al. 2016). Nitrate is neutralized by ammonium, potassium, and sodium.

Secondary sulfate source was identified by the third factor where the main indicators were SO_4_^2−^ and NH_4_^+^. This source is formed from agricultural NH_4_^+^ emissions together with SO_2_ from combustion of coal, biomass, and natural gas used for industry and domestic purposes. Secondary inorganic aerosols can be considered as regional scale pollution, long-range transport species, or anthropogenic pollution.

For the fourth factor, the main indicators are K^+^, Cu, Pb, and Fe, so it was related to biomass burning source. Manousakas declared the tracers for biomass burning as BC and K (Manousakas et al. 2015) or simply K (Almeida et al. 2005).

The fifth factor was identified as industry and/or soil source where the main indicators are Cr, Fe, PO_4_^3−^, Ca^2+^, and Na^+^. Viana et al. (2008) interpreted the tracers like Al/Si/Ca/Fe, Ti, Sr, and Mg as crustal matter (local or regional re-suspension, city dust, and road dust). Cr and Fe can be linked to industry, particularly to steel or non-ferrous metallurgical industry located in Krakow.

The traffic source was identified by the sixth factor where the main indicators are BC, Ti, Sr, Cu, Zn, and Mn. Viana associated vehicle source with the following markers: C, Fe, Ba, Zn, Cu, Cr, and Mn (Viana et al. 2008). Cr, Cu, and Zn are also recognized tracers for traffic (Manousaskas et al., 2015).

**Fig. 4:**
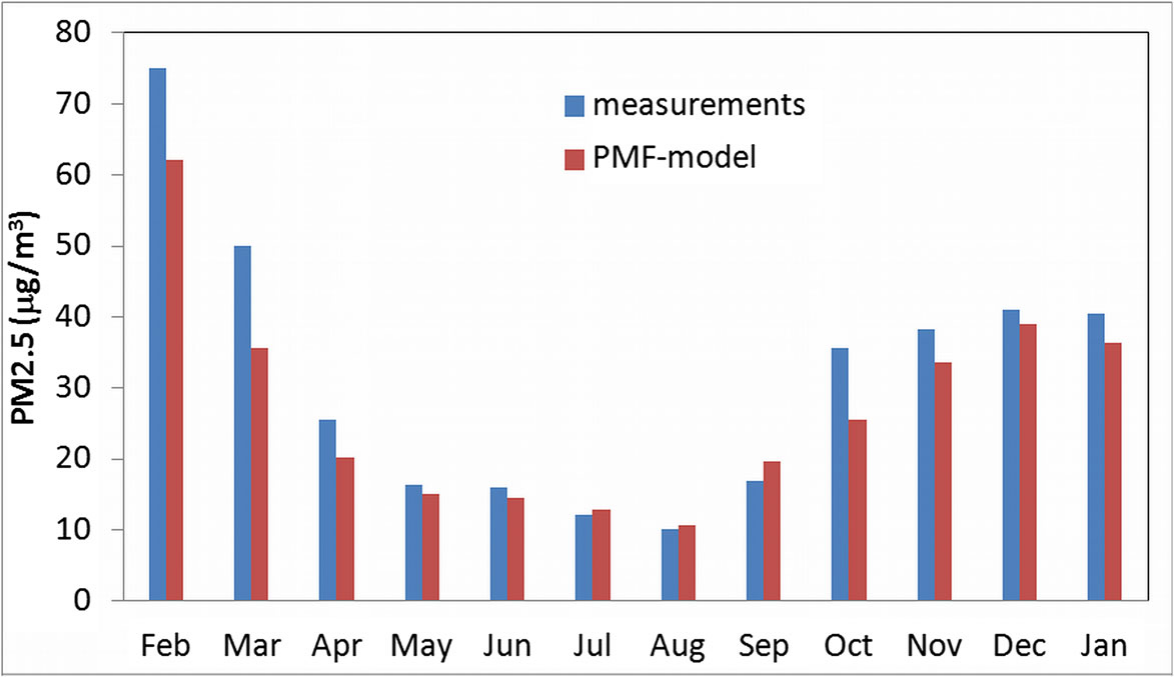
Modeled and measured monthly average concentrations of PM_2.5_

Mapping of “Fpeak Bootstrap” showed a strong stability (100%) of factors 1, 2, 3, and 5, while factors 4 and 6 had a good stability of 97%. In Fig. 4, monthly average concentrations of PM_2.5_, measured and predicted, are presented. The correlation between these values is 97%, but generally the predicted concentration is lower than that from the measurement. Probably, some sources have not been identified or the PMF modeling has delivered lower values then the real for some identified sources.

**Fig. 5:**
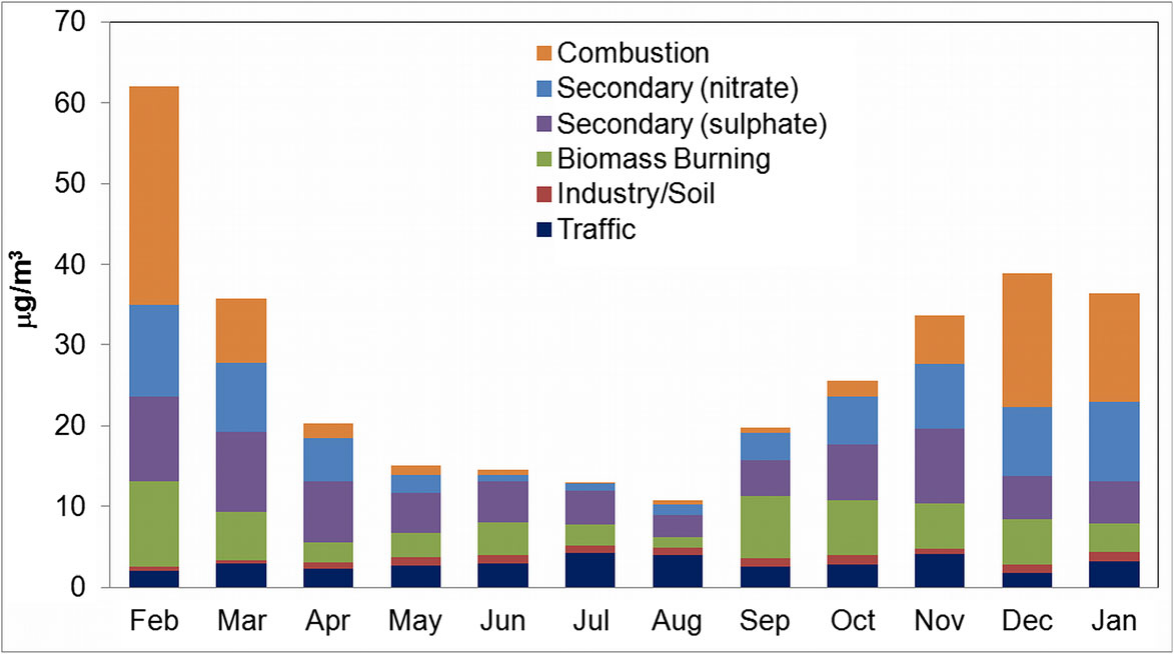
Distribution of source concentrations in PM_2.5_ modeled by PMF

Figure 5 presents the monthly contribution of PMF-resolved sources. The results illustrate that in several months of residential and domestic heating the dominating sources are combustion and secondary particulate matter. In January, contribution of combustion was equal to 27.2 μg/m^3^; secondary nitrate—11.4 μg/m^3^ and secondary sulfates—10.5 μg/m^3^. In summer months, the secondary sources have also significant percentage contribution to PM_2.5_ suggesting the presence of gases produced by combustion engines. In August, contribution of secondary sulfates was 2.7 μg/m^3^ (27% of PM_2.5_ mass) and secondary nitrates equal to 1.4 μg/m^3^ (14% of PM_2.5_ mass). The high rate of sulfate concentration in winter can be due to increased consumption of fossil fuels for winter heating during stagnation conditions. Similar results were presented by Jeong et al. in Edmonton (Jeong et al. 2011). Further studies are needed in order to determine local and regional sources of secondary aerosols. Biomass burning is used for domestic heating, and it is also used in a heating plant. This source is also correlated with seasons of heating having the contribution to PM_2.5_ of 1.4 μg/m^3^ in August and 10 μg/m^3^ in January. The traffic and industry and/or soil sources are rather stable—some observed differences can be related to weather conditions. Contribution of traffic was in the range of 1.6–4 μg m^−3^, dominating in July, August, and November. Small variability of 0.5–1.2 μg m^−3^ was apportioned to industry/soil sources. Similar periodic relations of traffic source were identified in other cities (Jeong et al. 2011; Amato et al. 2016; Yu et al. 2013). Annual source contributions to PM_2.5_ in Krakow (this study) and other cities are presented in Table 3. Our percentage results of source contribution are closest to that in Milan. Both cities are located in valleys, often shrouded in fog; surrounding mountains form a natural barrier that protects the cities from the major circulations so these might be the reasons for getting such results. In future, it will be interesting to perform wind direction and back trajectory analysis in order to tell apart local and regional sources—especially important in the case of secondary aerosols.

**Table 3:** Annual source contributions for PM2.5 [in %]

Factor no	Source category	Krakow this study	Erfurt^a^	Barcelona^b^	Florence^b^	Milan^b^	Edmonton^c^ Canada	Beijing^d^ China	Central and eastern Europe^e^
F-1	Combustion	22.9		5	6			16	32
F-2	Secondary nitrate	17.1	39	13	14	30	21.9		
F-3	Secondary sulfate	19.3		38	30	19	19	26.5	
F-4	Biomass burning	15.6			20	17	12	11.2	
F-5	Industry/soil	2.5	15	9 + 10	5	5	4.9	6 + 10.4	16
F-6	Traffic	8.3	36	19 + 1	18 + 2	6 + 8	12.4	17.1 + 12.7	19
	Non-identified	14.3	10	5	5	9			16

^a^Viana et al. (2008)

^b^Amato et al. (2016)

^c^Jeong et al. (2011)

^d^Yu et al. (2013)

^e^Karagulian et al. (2015)

## Conclusions

Aerosol chemical composition data, collected for a 1-year period (February 2014–January 2015) in Krakow, were analyzed to identify seasonal variation and estimate chemical species contributions to the PM_2.5_ mass. Highest monthly ion variability was for nitrates (from 0.07 to 4.8 μg m^−3^), Cl^−^ (from 0.06 to 3.8 μg m^−3^), NH_4_^+^ (from 0.62 to 4.26 μg m^−3^), and sulfates (from 1.5 to 5.4 μg m^−3^). As far as elements are concerned, the highest variability was observed for Pb (from 4 to 60 ng m^−3^), Br (from 2 to 32 ng m^−3^), Zn (from 12 to 164 ng m^−3^), and Cu (from 2 to 17 ng m^−3^).

The same data were analyzed using the PMF model to identify and apportion the most probable sources affecting the PM_2.5_ mass. Six sources were identified: secondary sulfate, secondary nitrate, combustion, biomass burning, steel industry/soil dust, and traffic. Highest seasonal variation was observed for the combustion source (from 0.17 to 27 μg m^−3^)—in winter months, the contribution of this source to total PM_2.5_ mass was about 40%. Biomass burning, secondary sulfate and nitrate sources also owned big seasonal variation having in summer concentrations ten times lower than in winter. These four sources have dominated contribution to total PM_2.5_ mass by 88 to 96% during the winter season. In summer, this rate was about 60%. Industry/soil dust and traffic sources had no significant seasonal variation.

The results presented in this work may have an impact on air pollution reduction strategies in Krakow and, as a result, protect human health. It can be assumed that similar PM sources and their seasonal variations occur in other urban areas of Poland. Of course, finding the main sources is a far cry from resolving the pollution problem. That would require a detailed understanding of atmospheric transport phenomena, a knowledge of trans-boundary contributions to the PM as well as an inter-comparison of results. However, the reported data add to a map of main pollutants across Europe.
